# Detection of *Sarcocystis halieti* in muscles of raptors from Lithuania

**DOI:** 10.3389/fvets.2025.1568013

**Published:** 2025-07-22

**Authors:** Petras Prakas, Tautvilė Šukytė, Evelina Juozaitytė-Ngugu, Dalius Butkauskas

**Affiliations:** Laboratory of Molecular Ecology, Nature Research Centre, Vilnius, Lithuania

**Keywords:** *Sarcocystis*, Accipitriformes, Strigiformes, methylene blue staining, molecular identification, *ITS1*

## Abstract

**Background:**

The genus *Sarcocystis* comprises a diverse group of apicomplexan parasites that infect reptiles, birds, and mammals. They are characterized by the formation of sarcocysts in the muscles of the intermediate host and the development of sporocysts in the intestines of the definitive host. Raptors usually act as definitive hosts for numerous *Sarcocystis* species; however there is a lack of studies on *Sarcocystis* in the muscles of raptorial birds. Therefore, we aimed to assess infection rates and identify *Sarcocystis* species in the muscles of raptors in Lithuania.

**Methods:**

Muscle samples from 90 raptors (Accipitriformes, Falconiformes, and Strigiformes) were collected throughout Lithuania and analyzed for *Sarcocystis* spp. Sarcocysts isolated from fresh methylene blue-stained muscle samples were identified using the internal transcribed spacer region 1 sequence genetic marker.

**Results:**

Under the light microscope, sarcocysts were detected in 8.9% (8/90) of the raptors examined. Sarcocysts were found in the leg muscles of common buzzards (*Buteo buteo*), tawny owls (*Strix aluco*), and a long-eared owl (*Asio otus*); neck muscles of a Eurasian goshawk (*Accipiter gentilis*), rough-legged buzzard (*Buteo lagopus*), and long-eared owl; and thoracic muscles of a rough-legged buzzard. We observed no sarcocysts in the cardiac muscles. Representatives of one *Sarcocystis* species, *S*. *halieti* were molecularly identified in seven birds.

**Conclusion:**

This is the first study to report five new intermediate hosts for *S*. *halieti*. Further investigations are needed to assess the possible pathogenicity of *S*. *halieti* in extra-intestinal organs of raptors.

## 1 Introduction

Over the last 500 years, 140 species of birds have become extinct, more than any other vertebrate group (1). Over half of raptors are seeing population reductions worldwide, and 18% of raptors are in danger of going extinct (2). The most common causes of morbidity in raptors are infectious and parasitic diseases, traumatic injuries, toxicosis, and metabolic or nutritional diseases (3). Raptors feature a large diversity of protozoan, helminth, and associated bacterial infections of the digestive system. For instance, the study conducted in 2000 has shown that even 89.2% of all raptors were infected with helminths such as Acanthocephala, Cestoda, Nematoda, and Trematoda (4). In addition, in the same study, coccidians were found in 31.4% of raptors studied. In general, there is still a lack of knowledge about parasite infections in the muscles of raptors (5, 6).

*Sarcocystis* spp. are apicomplexan intracellular parasites that have a mandatory two-host life cycle. These parasites are known for their characteristics of forming sarcocysts in the muscles of various animals, including poikilothermic ones, birds, and mammals. Two routes of *Sarcocystis* spp. infection are possible. The primary transmission route is fecal-oral, involving the ingestion of water or food contaminated with sporocysts/oocysts from the feces of the definitive host (DH). The second mode of transmission is ingestion of muscles or other tissues of intermediate hosts (IH) that harbor mature sarcocysts (7).

Globally, various bird species serve as hosts for more than 48 *Sarcocystis* species (8). Among these, 32 are named species described as utilizing birds as IHs. In contrast, at least 16 *Sarcocystis* species have been molecularly confirmed to use birds as DHs (6, 9, 10). *Sarcocystis* spp. are mainly asymptomatic for DHs, while these parasites can cause histopathological changes for tissues of IHs (11). Three species, *Sarcocystis halieti*, *Sarcocystis falcatula*, and *Sarcocystis wobeseri*, have been found in extra-intestinal tissues of birds belonging to orders Accipitriformes and Strigiformes (6, 10–15). *Sarcocystis falcatula* is highly pathogenic and can cause lung injuries and encephalitis, which can be fatal even to birds (8, 10, 11, 16). However, the prevalence of *S*. *falcatula* is restricted to North and South America, where their host species belonging to the genus *Didelphis* are distributed (7, 17, 18). *Sarcocystis halieti* is considered to be pathogenic due to a report of severe multifocal granulomatous encephalitis in the little owl (*Athene noctua*) (10). This *Sarcocystis* species is multi-host specific and has been identified in Old and New World birds (6, 10, 15, 19). Sarcocysts of *S*. *wobeseri* have been identified in muscles of Anseriformes, Charadriiformes, and Accipitriformes (14, 20, 21); however, the pathogenicity of this *Sarcocystis* species has not yet been fully investigated. Based on molecular analysis, DHs of *S*. *halieti* and *S*. *wobeseri* belong to the order Accipitriformes (19, 22–25).

In Lithuania, the muscles of birds belonging to Anseriformes, Charadriiformes, and Passeriformes have been the most extensively studied, while raptors have not yet been investigated for muscular sarcocystosis. Thus, our study aimed to evaluate infection rates and identify *Sarcocystis* species found in the muscles of raptors from Lithuania.

## 2 Materials and methods

### 2.1 Sample collection

Muscle samples of 90 raptorial birds belonging to orders Accipitriformes, Falconiformes, and Strigiformes were collected in different regions of Lithuania (54–55°N, 21–24°W) between 2014 and 2024 and examined for the presence of sarcocysts of *Sarcocystis* spp. (Table 1). Samples of birds were retrieved from Kaunas Tadas Ivanauskas Zoology Museum, which is the national authority that runs wildlife research programs and is responsible for monitoring dead animals found. Birds used for our study were killed along roadsides as well as after collisions with architectural structures and high-voltage wires. Muscle samples were kept frozen (−20°C) until microscopic examination of *Sarcocystis* parasites. The conducted study was approved by the Animal Welfare Committee of the Nature Research Center (no. GGT-9). It should be noted that only some muscle types were available for each bird examined. A total of 243 muscle samples, 54 leg muscle samples, 53 neck tissue samples, 83 thoracic muscles samples and 53 heart samples were tested for sarcocysts (Table 1).

**Table 1:** The infection rates of *Sarcocystis* spp. in various muscles of raptors.

Bird order	Bird species	No. of birds infected	No. of birds examined	Prevalence, %	No. of birds infected/examined in different muscles			
					Leg	Neck	Thoracic	Heart
Accipitriformes	Eurasian goshawk (*Accipiter gentilis*)	1	15	6.7	0/12	1/12	0/15	0/12
Accipitriformes	Eurasian sparrowhawk (*Accipiter nisus*)	0	11	0	0/8	0/7	0/10	0/6
Accipitriformes	Common buzzard (*Buteo buteo*)	2	23	8.7	2/16	0/14	0/21	0/14
Accipitriformes	Rough-legged buzzard (*Buteo lagopus*)	1	2	50	–	1/2	1/1	–
Accipitriformes	Western marsh harrier (*Circus aeruginosus*)	0	1	0	0/1	0/1	0/1	0/1
Strigiformes	Long-eared owl (*Asio otus*)	2	12	16.7	1/7	1/7	0/12	0/7
Strigiformes	Eurasian pygmy owl (*Glaucidium passerinum*)	0	3	0	–	–	0/1	0/2
Strigiformes	Tawny owl (*Strix aluco*)	2	22	9.1	2/10	0/10	0/21	0/11
Falconiformes	Common kestrel (*Falco tinnunculus*)	0	1	0	–	–	0/1	–

### 2.2 Microscopical identification of *Sarcocystis* spp

*Sarcocystis* spp. infection was confirmed by microscopy of stained muscle samples (leg, neck, thoracic, and heart). Twenty-eight rice-sized pieces of each muscle (1 g ± 0.3) were cut from each bird, stained with 0.2% methylene blue for 35 min, soaked in 1.5% acetic acid for 25 min, squeezed into a glass compressor, and examined under light microscopy (LM) at a magnification of × 200. Infection intensity was quantified by counting sarcocysts within 28 tissue sections obtained from the muscle. The sarcocysts detected were removed with a preparation needle, each placed separately in a 1.5 mL microcentrifuge tube containing 1 mL of 96% ethanol and stored at 4°C. The ethanol was replaced once a week for 1 month or until the blue color faded.

The bird muscle samples in which *Sarcocystis* spp. infection was detected by methylene blue-staining were re-examined in fresh squashed preparations using a 0.9% NaCl solution. Observed sarcocysts were morphologically characterized at × 200 and × 400 magnifications. The discovered sarcocysts were free from myofibrils as much as possible, and a part of the bradyzoites was released by pressing the cyst firmly. The excreted sarcocysts were placed in individual microcentrifuge tubes filled with 96% ethanol and stored at −20°C until DNA isolation.

### 2.3 Molecular analysis of detected sarcocysts

The genomic DNA from each isolated sarcocyst was purified using the GeneJET Genomic DNA Purification Kit (Thermo Fisher Scientific Baltics, Vilnius, Lithuania) according to the manufacturer’s instructions. Obtained DNA samples were kept frozen at −20°C for further molecular analysis. *Sarcocystis* species identification was performed using the internal transcribed spacer 1 (*ITS1*) region, which was previously shown to be variable enough and could be used for the discrimination of avian *Sarcocystis* species (26).

Firstly, DNA fragments were amplified using nested PCR. Each reaction was carried out using DreamTaq PCR Master Mix (Thermo Fisher Scientific Baltics, Vilnius, Lithuania). In the first step, a forward NITSpauk1 (5′-TGTCCGGAATGGGAAGTTTT-3′) and reverse NITSpauk2 (5′-ACACCATCCDAAATTCTCAG-3′) primers were used. In the second step, the primer pair NITSpauk3 (5′-GGAAGGATCATTCACACGTT-3′)/NITSpauk4 (5′- ATCACTGCAAGTTCCAACCA-3′) was used (11). After a second round of nested PCR, the resulting 261 bp-long products were purified and sequenced as previously specified (11). Based on the short *ITS1* fragments, *S*. *halieti* was detected in all cases of successful amplification and sequencing.

For the conclusive *Sarcocystis* species identification we used longer *S*. *halieti* specific primers, forward GsShalF1 (5′-GATAATTGACTTTACGCGCCATTAC-3′) and reverse GsShalR2 (5′-CCATCCCTTTTTCTAAAGGAGGTC-3′) (27) amplifying about a 645 bp-long *ITS1* fragment. The generation of such fragments was carried out in two alternative ways, by using nested or direct PCR. In case of the nested PCR, SU1F/5.8SR2 (5′- GATTGAGTGTTCCGGTGAATTATT-3′/5’-AAGGTGCCATTTGCGTTCAGAA-3′) external primers (26) and DreamTaq PCR Master Mix were used following previously described procedures (27). Direct PCR was performed using PlatinumTM SuperFi II Green PCR Master Mix (Thermo Fisher Scientific Baltics, Vilnius, Lithuania). PCR cycling conditions were as follows: initial denaturation for 30 s at 98°C, 35 cycles of 10 s at 98°C, 10 s at 60°C, 30 s at 72°C, and final extension for 5 min at 72°C. Visualization and purification of PCR products and Sanger sequencing were performed as described in Prakas et al. (11).

### 2.4 Phylogenetic analysis

The obtained *ITS1* sequences (GenBank accession numbers: PQ900168–PQ900180) were compared with those of various *Sarcocystis* spp. using nBLAST sequence similarity search algorithm (28). Phylogenetic analyses were carried out with the help of MEGA v. 11.0.13 software (29). Multiple sequence alignments were generated using the MUSCLE algorithm. Phylogenetic trees were constructed using the Maximum Likelihood (ML) method. The nucleotide substitution model that best suited the analyzed alignments was selected using the MEGA “Find Best DNA/Protein Models (ML).” The robustness of phylogeny was tested using the bootstrap method with 1,000 bootstrap replications.

## 3 Results

### 3.1 Infection rate and parasite load of sarcocysts in raptors

The infection rate of *Sarcocystis* spp. was established by examining methylene blue-stained muscle samples. Sarcocysts were detected in 8.9% (8/90) of muscles of Lithuanian raptors examined (Table 1). Sarcocysts were found in the leg muscles of two common buzzards (*Buteo buteo*) (12.5%, 2/16) and tawny owls (*Strix aluco*) (20%, 2/10), and one long-eared owl (*Asio otus*) (14.3%, 1/7). In addition, *Sarcocystis* spp. were found in the neck muscles of a single Eurasian goshawk (*Accipiter gentilis*) (8.3%, 1/12), rough-legged buzzard (*Buteo lagopus*) (1/2), and long-eared owl (14.3%, 1/7). Furthermore, *Sarcocystis* spp. have been observed in the thoracic muscles of one rough-legged buzzard (Table 1). No sarcocysts were observed in the hearts of birds examined. Overall, of the 243 muscle samples stained with methylene blue, sarcocysts were found in nine of them (3.7%).

Higher, but statistically not significant (χ2 = 0.26, *p* = 0.612), infection rate was identified in birds of the order Strigiformes (4/37; 10.8%) compared to that determined in birds of the order Accipitriformes (4/52; 7.7%). Most frequently, sarcocysts were detected in leg muscles (5/54; 9.3%), followed by neck (3/54; 5.6) and thoracic (1/83; 1.2) muscles.

The parasite load ranged from one to 15 cysts/g of muscles stained with methylene blue; the mean and median parasite load were two and four cysts/g of muscle, respectively.

### 3.2 Morphology of detected sarcocysts

**Figure 1:**
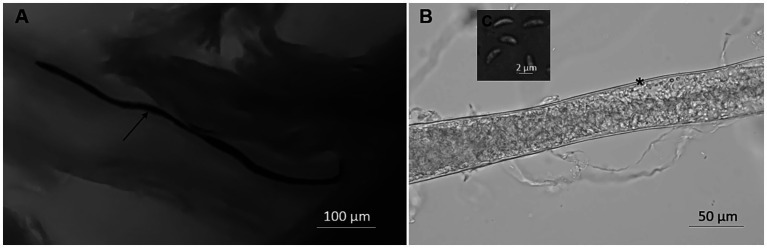
Morphology of *Sarcocystis halieti* in tissue sample taken from leg muscle of raptors. **(A)** Sarcocyst from common buzzard (*Buteo buteo*) stained with methylene blue; the arrow shows the cyst. **(B)** Fragment of the smooth cyst wall in fresh preparation from tawny owl (*Strix aluco*); *marked the cyst wall. **(C)** Banana-shaped bradyzoites in fresh sample from tawny owl.

Sarcocyts were observed in fresh muscle samples from only two birds. In methylene blue-stained samples, *Sarcocystis* spp. sarcocysts were found to be 4,952 × 34 μm (range: 2673–7,380 × 25–64 μm; *n* = 10) in size and looked like tiny threads (Figure 1A). The sarcocyst wall found in unstained squashed muscles appeared to be smooth and 1 μm thick (Figure 1B). Septa split sarcocysts into sections that were filled with 6.7 × 1.9 μm (4.9–8.7 × 1.2–2.5 μm; *n* = 7) banana-shaped bradyzoites (Figure 1C).

### 3.3 Molecular identification of *Sarcocystis halieti*

In fresh samples, sarcocysts of *Sarcocystis* spp. were only observed and isolated from neck muscles of the rough-legged buzzard and leg muscles of the tawny owl. In eight more *Sarcocystis* spp. positive samples, sarcocysts were found only in methylene blue-stained preparations. One sarcocyst from each *Sarcocystis* spp. positive sample was subjected to molecular analysis. Using the *Sarcocystis* spp.-specific NITSpauk3/NITSpauk4 primer pair, we obtained three different 221 bp-long *ITS1* genotypes differing in 1–2 SNPs (single nucleotide polymorphisms). These *ITS1* sequences showed 95.9–100% identity to those of *S*. *halieti*, 94.6–95.0% similarity to that of *Sarcocystis* sp. isolate Skua-2016-CH (MW160469), 94.1–94.6% similarity to those of *Sarcocystis* sp. isolate 38P (PQ133336) and *Sarcocystis* sp. ex *Accipiter cooperii* (KY348755), similarity to that of 93.6–94.1% *Sarcocystis* sp. ex *Corvus corax* (MZ707151) and 92.9–93.3%% similarity to that of *Sarcocystis corvusi* (JN256119).

Using *S*. *halieti*-specific primers (GsShalF1/GsShalR2), we were able to amplify five samples when conventional PCR and PlatinumTM SuperFi polymerase were applied, whereas three samples were positive when nested PCR and DreamTaq polymerase were used (Table 2). The five 596 bp-long *ITS1* sequences generated represented four distinct genetic variants that differed in one to three SNPs. The comparison of these 596 bp-long *ITS1* sequences demonstrated 96.8–100% identity to those of *S*. *halieti*, 96.1–96.5% similarity to that of *Sarcocystis* sp. isolate Skua-2016-CH, 94.6–95.0% similarity to that of *Sarcocystis* sp. ex *Corvus corax*, 92.8–93.2% similarity to that of *Sarcocystis* sp. isolate 38P, 92.7–93.0% similarity to that of *Sarcocystis* sp. ex *Accipiter cooperii*, 91.7–92.3% similarity to those of *S*. *columbae*, and 90.9–91.2% similarity to that of *S*. *corvusi*. Thus, based on DNA sequence analysis *S*. *halieti* was identified in eight muscle samples of raptors from Lithuania (Table 2).

**Table 2:** Identification of *Sarcocystis halieti* in muscles of raptors using different muscle examination techniques, PCR methods and polymerases.

Bird species	ID	Muscle	Fresh/stained	Nested PCR	Nested PCR	Conventional PCR
				DreamTaq	DreamTaq	PlatinumTM SuperFi
				NITSpauk3/NITSpauk4	GsShalF1 GsShalR2	GsShalF1/GsShalR2
Eurasian goshawk	9	Neck	Stained	PQ900173	Neg.	PQ900168
Common buzzard	5	Leg	Stained	PQ900174	*	PQ900169
Common buzzard	19	Leg	Stained	Neg.	Neg.	Neg.
Rough-legged buzzard	2	Thoracic	Stained	PQ900175	Neg.	Neg.
Rough-legged buzzard	2	Neck	Fresh	PQ900176	*	PQ900170
Long-eared owl	9	Thoracic	Stained	PQ900177	Neg.	PQ900171
Long-eared owl	11	Neck	Stained	PQ900178	Neg.	Neg.
Tawny owl	18	Leg	Stained	PQ900179	Neg.	Neg.
Tawny owl	30	Leg	Fresh	PQ900180	*	PQ900172

ID, identification number of bird sample; Neg., not amplified sample. *positive PCR, but not sequenced.

### 3.4 Phylogenetic results

**Figure 2:**
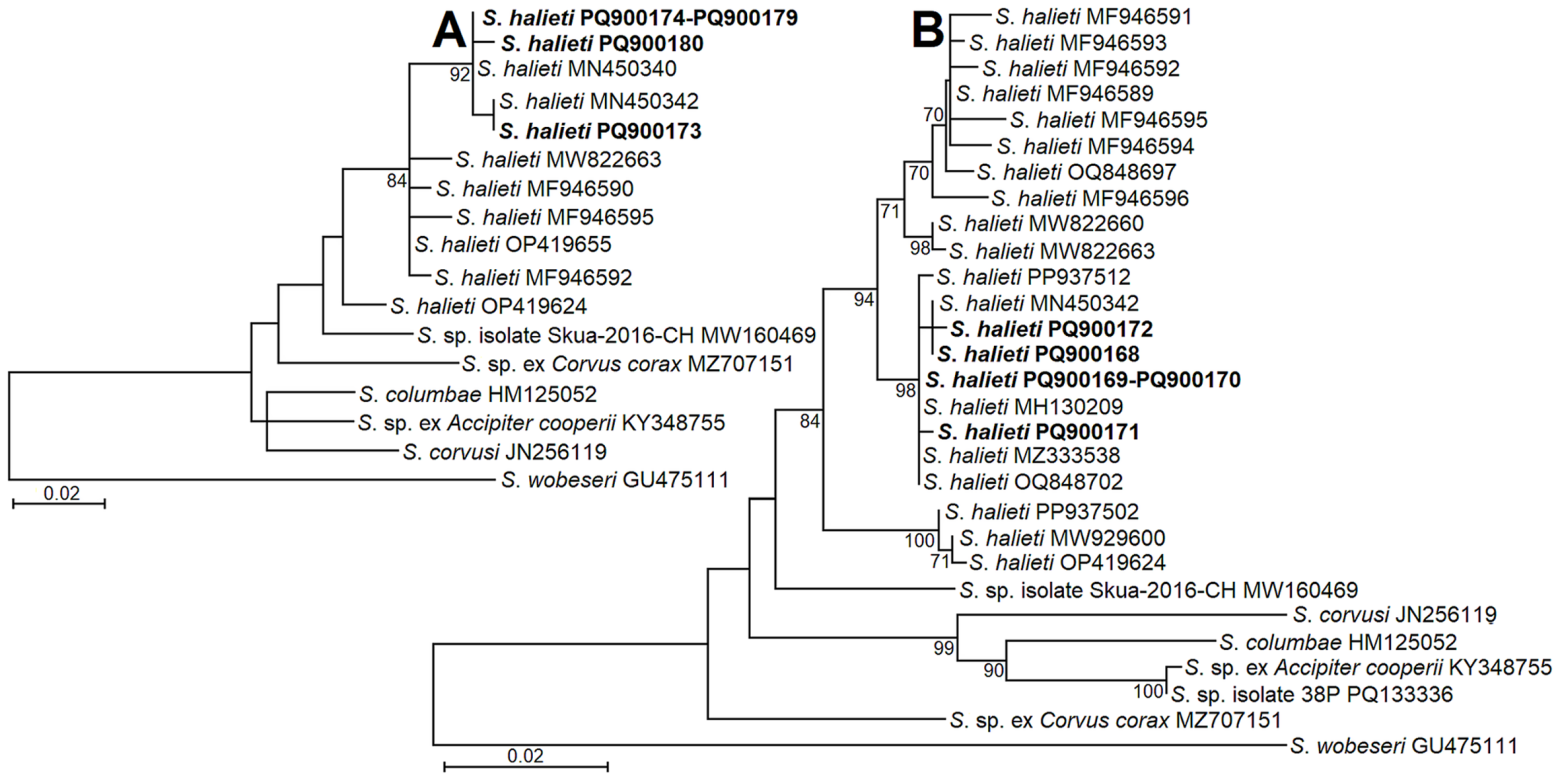
The phylogenetic placement of *S. halieti* isolates obtained from raptors in Lithuania. Phylogenetic trees were obtained analyzing *ITS1* sequences obtained with the help of NITSpauk3/NITSpauk4 **(A)** and GsShalF1/GsShalR2 **(B)** primer pairs. Trees were constructed using ML method, scaled according to the branch length and rooted on *S*. *wobeseri*. In the left analysis **(A)** the alignment consisted of 17 sequences and 225 nucleotide positions; Tamura 3 parameter + G nucleotide substitution model was set. In the right analysis **(B)** the alignment contained of 29 sequences and 624 nucleotide positions; HKY nucleotide substitution model was set. Sequences generated in present study are indicated in boldface.

For the phylogenetic analyses, all possible different genetic variants of *S*. *halieti* taken from GenBank were included. In addition to our *S*. *halieti* sequences, eight and 18 different *ITS1* genotypes were analyzed, when sequences were obtained using NITSpauk3/NITSpauk4 and GsShalF1/GsShalR2 primers, respectively. Furthermore, sequences of other *Sarcocystis* spp. showing the highest similarity to our sequences were used to reveal phylogenetic relationships. The *ITS1* sequences generated in the present study with fairly high bootstrap support values (84 and 94) were grouped with other sequences of *S*. *halieti* (Figure 2). However, using short 221 bp-long *ITS1* fragments, a single sequence of *S*. *halieti* OP419624 formed a separate branch in the phylogram (Figure 2A). Based on longer ~596 bp-long *ITS1* sequences, *S*. *halieti* genetic variants clustered into several clades (Figure 2B). Sequences obtained in the present work were grouped with those of *S*. *halieti* isolated from various IHs (birds classified as insectivorous, piscivores, omnivores, and raptors) and DHs of the order Accipitriformes collected in the Czeck Republic, Lithuania, and Spain. Both phylogenetic analyses showed that *S*. *halieti* was most related to *Sarcocystis* sp. from Chilean skua (*Stercorarius chilensis*) (MW160469).

## 4 Discussion

In the current study, we provide the first data on *Sarcocystis* in the muscles of raptors from Lithuania. Based on methylene blue-staining, sarcocysts of *Sarcocystis* spp. were observed in 8.9% of 90 muscle samples of raptorial birds from Lithuania (Table 1). Sarcocysts were found in the leg, neck, and thoracic muscles of the birds studied, but not in the heart. Using the same method of microscopic analysis, birds of three families, i.e., Anatidae, Corvidae, and Laridae, were the most extensively studied for the presence of sarcocysts in Lithuania (20, 21, 30–36). Comparing the estimated infection rates, sarcocysts were much less frequently found in the muscles of raptors than in those of corvids (62/181, 34.3%), anatids (100/342, 29.2%), and larids (21/140, 15.0%) (20, 21, 36, 37).

It should also be mentioned that there is also a lack of comprehensive research on *Sarcocystis* in extra-intestinal tissues worldwide (6). Previously, *Sarcocystis* spp. were detected in the skeletal muscles, heart, and brain of raptors (5, 6, 10, 12, 13, 15, 38). In general, the observed infection rates of *Sarcocystis* spp. in muscles highly depend on the method applied (6), methylene blue-staining, histological examination, muscle digestion, or immunofluorescence antibody testing (5, 39–41). However, in most cases the infection rates observed in the muscles of raptors were in the range of 5 and 15%, despite studies having been carried out in different geographical regions, Australia, Brazil, Germany, Kazakhstan, and Spain (5, 6, 39, 41, 42).

By microscopical examination, thin and smooth sarcocysts, similar to those found in our study (Figure 1), were also detected in muscles of common kestrel (*Falco tinnunculus*) from Kazakhstan (42), in common buzzard from Germany (5), and in several species of raptors from the United States (38). However, several avian *Sarcocystis* spp. form sarcocysts with smooth and thin cyst walls. Furthermore, two *Sarcocystis* species (*S*. *halieti* and *S*. *wobeseri*) having thin sarcocysts walls have been confirmed in raptors (6, 14). Therefore, conclusive identification of sarcocysts found in raptors’ muscles requires molecular analysis methods.

In the present study, by using nested PCR and NITSpauk3/ NITSpauk4 primers, *S*. *halieti* was confirmed in muscles of seven birds belonging to five different species of orders Accipitriformes and Strigiformes (Table 2). Based on a comparison of *ITS1* sequences, the obtained intraspecific and interspecific genetic variability values did not overlap, showing that the species was correctly identified. The detection of *S*. *halieti* in the samples analyzed was also confirmed by phylogenetic analysis, as investigated parasite isolates significantly clustered with other isolates of *S*. *halieti* (Figure 2). Thus, relatively small 221 bp-long and 596 bp-long fragments of *ITS1* can be used for the identification of *S*. *halieti*. *Sarcocystis* spp. from birds have evolved more recently compared to species found in mammals and reptiles (7). Therefore, conservative markers, such as ribosomal *18S* rRNA, the mitochondrial cytochrome c oxidase subunit 1 gene (*cox1*), and the RNA polymerase B gene of the apicoplast genome (rpoB), are not variable enough to discriminate all *Sarcocystis* species with birds as IH (21, 24, 26, 43). By contrast, numerous studies have confirmed that highly variable *ITS1* is currently the most appropriate marker for distinguishing avian *Sarcocystis* species (6, 15, 21, 23, 36, 41, 43). In the current work, the GsShalF1/GsShalR2 primer pair has been confirmed in several studies to be specific for the amplification of *S*. *halieti* (25, 27). Other primers (internal NITSpauk1/NITSpauk2 and external NITSpauk3/NITSpauk4) we have applied in the present study were theoretically designed for the detection of *Sarcocystis* spp. employing birds and predatory mammals as their IHs (11). Due to short products amplified and due to *Sarcocystis* spp. conservative primers NITSpauk1, targeting *18S* rRNA, the above-described primers can be used for the initial screening of muscles of birds and predatory mammals for the presence of *Sarcocystis* spp.

Based on *ITS1* or *28S* rRNA *S*. *halieti* was previously detected in muscles and brains of birds belonging to even six orders: Accipitriformes, Charadriiformes, Passeriformes, Procellariiformes, Strigiformes, and Suliformes (6, 10, 11, 15, 20, 21, 23, 36, 41, 43). Sarcocysts of *S*. *halieti* were found in birds from Europe, South America, and Asia (6, 11, 15, 20, 41; also see PQ270246 and PQ276104 GenBank records). In this study, we provide new host records of *S*. *halieti* in common buzzard, Eurasian goshawk, rough-legged buzzard (Accipitridae), long-eared owl and tawny owl (Strigidae). During this study, the material obtained from Kaunas Tadas Ivanauskas Zoology Museum was frozen, which prevented us from performing histopathological examinations. Prior to this study, *S*. *halieti* was confirmed in Lithuania in muscles of birds of Corvidae, Laridae, and Phalacrocoracidae families (20, 21, 36, 43). Furthermore, relatively high detection rates of *S*. *halieti* were established in intestinal scrapings of common buzzards, Eurasian goshawks and Eurasian sparrowhawks (*Accipiter nisus*) collected in Lithuania (24, 25). Therefore, we can expect to find this *Sarcocystis* species in understudied IH. According to the current knowledge, *S*. *halieti* is widely adapted to parasitise New World and Old World birds that live in terrestrial and aquatic environments and are prey or predators (6, 10, 11, 15, 20, 21, 23, 36, 41, 43). It is worrisome that *S*. *halieti* is thought to cause granulomatous encephalitis in little owl (10), but most of the studies that have been done so far have only looked at how to diagnose this parasite in muscle tissues (20, 21). Furthermore, neurological sarcocystosis was reported in several raptors, i.e., American goshawk (*Astur atricapillus*), bald eagle (*Haliaeetus leucocephalus*), and golden eagle (*Aquila chrysaetos*) from the United States (reviewed by 7). Thus, future extensive histopathological examinations of various birds are desirable to enclose pathogenicity and threat of *S*. *halieti* and potentially other *Sarcocystis* species parasitizing raptors.

To detect sarcocysts, we used a modified microscopic-compressor method initially designed to examine muscle samples of *Trichinella* spp. (44, 45). The method based on the analysis of methylene blue-stained muscle samples squeezed between glass compressors is superior to the analysis of fresh muscle samples in the case of low parasite loads of *Sarcocystis* spp. (46). Furthermore, in the case of our study, we obtained intact sarcocysts, which is not possible in the case of tissue digestion, a highly effective method for detecting low levels of *Sarcocystis* infection in muscle samples analyzed (47). Extraction of high-quality DNA from stained and/or fixed samples remains challenging (48). The fixation of tissues leads to fragmentation of the nucleic acids and has a negative impact on the quantity of recovered DNA. A lot of studies have been performed to optimize the extraction process from formalin-fixed paraffin-embedded (FFPE) samples (49). Several studies have successfully identified *Sarcocystis* species using molecular techniques from FFPE samples (12, 50, 51). Some of the studies have shown that PCR primers targeting relatively short DNA fragments (200–500 bp) facilitate the amplification of *Sarcocystis* species DNA from FFPE samples (11, 51). Initially, for the screening of *Sarcocystis* spp. in methylene blue samples, we selected to use a nested PCR approach. This PCR method is an attractive choice because it increases sensitivity and specificity and can amplify target sequences presented in low abundance (52, 53). Research has demonstrated the value of nested PCR in amplifying DNA fragments from FFPE samples (11, 54). However, nested PCR has the disadvantage of increasing the number of false positive results, mainly due to cross-contamination caused by the transfer of the products of the first amplification step to a second tube (55). Therefore, we subsequently tried direct PCR with *S*. *halieti*-specific primers. Based on direct PCR, 1/7 and 3/7 methylene blue samples were successfully amplified using standard and high-fidelity polymerases, respectively (Table 2). In each case, the Sanger sequencing confirmed *S*. *halieti* in PCR-positive samples. To the best of our knowledge, this study is the first successful attempt to identify *Sarcocystis* species by PCR-based methods from methylene blue-stained muscle samples. The applied method is attractive since *Sarcocystis* species are diagnosed from microscopically observed sarcocysts rather than from bradyzoite suspension obtained after digestion or from DNA extracted from host tissue (47, 56). We suggest this method when the muscle samples have been frozen for too long and when parasite load is low. However, optimisation of DNA extraction and PCR procedures from sarcocysts stained with methylene blue is still needed.

## 5 Conclusion

The present study is the first attempt to detect *Sarcocystis* parasites in the muscles of raptors from Lithuania. Based on methylene blue-staining, sarcocysts were detected in 8.9% (8/90) of the raptors tested, which is consistent with the low prevalence of *Sarcocystis* spp. in raptors reported worldwide. Using DNA sequence comparisons, the potentially pathogenic *S*. *halieti* was identified in five new intermediate host species. Due to its pathogenic potential, further extensive histopathological examinations of these hosts are recommended. In addition, for the first time, we managed to identify *Sarcocystis* species from methylene blue-stained muscle samples. We recommend using the suggested method when the available tissue samples are not fresh, or parasite load is low.

## Data Availability

The original data are available in a publicly accessible repository of the GenBank under the accession numbers: PQ900168–PQ900180.
